# Supplementary material for: A fault-intrusion-tolerant system and deadline-aware algorithm for scheduling scientific workflow in the cloud
Source: PeerJ Comput Sci. 2021 Nov 2;7:e747. doi: 10.7717/peerj-cs.747 (PMC8576554; doi:10.7717/peerj-cs.747)
Supplement: Supplemental Information 6 [file peerj-cs-07-747-s006.zip › Myproject1.1/docs/index-files/index-17.html]

R-Index


JavaScript is disabled on your browser.


- Overview
- Package
- Class
- Use
- Tree
- Deprecated
- Index
- Help

- Prev Letter
- Next Letter

- Frames
- No Frames

- All Classes

A B C D E F G H I J L M N O P Q R S T U V W Z 


## R

RamProvisioner - Class in org.cloudbus.cloudsim.provisioners
:   RamProvisioner is an abstract class that represents the provisioning policy of memory to virtual
    machines inside a Host.

RamProvisioner(int) - Constructor for class org.cloudbus.cloudsim.provisioners.RamProvisioner
:   Creates the new RamProvisioner.

RamProvisionerSimple - Class in org.cloudbus.cloudsim.provisioners
:   RamProvisionerSimple is an extension of RamProvisioner which uses a best-effort policy to
    allocate memory to a VM.

RamProvisionerSimple(int) - Constructor for class org.cloudbus.cloudsim.provisioners.RamProvisionerSimple
:   Instantiates a new ram provisioner simple.

RandomConstants - Class in org.cloudbus.cloudsim.examples.power.random
:   If you are using any algorithms, policies or workload included in the power package please cite
    the following paper:
    Anton Beloglazov, and Rajkumar Buyya, "Optimal Online Deterministic Algorithms and Adaptive
    Heuristics for Energy and Performance Efficient Dynamic Consolidation of Virtual Machines in
    Cloud Data Centers", Concurrency and Computation: Practice and Experience (CCPE), Volume 24,
    Issue 13, Pages: 1397-1420, John Wiley & Sons, Ltd, New York, USA, 2012

RandomConstants() - Constructor for class org.cloudbus.cloudsim.examples.power.random.RandomConstants


RandomHelper - Class in org.cloudbus.cloudsim.examples.power.random
:   The Helper class for the random workload.

RandomHelper() - Constructor for class org.cloudbus.cloudsim.examples.power.random.RandomHelper


RandomPlanner - Class in org.workflowsim.planning
:   The Random planning algorithm.

RandomPlanner() - Constructor for class org.workflowsim.planning.RandomPlanner


RandomRunner - Class in org.cloudbus.cloudsim.examples.power.random
:   The example runner for the random workload.

RandomRunner(boolean, boolean, String, String, String, String, String, String) - Constructor for class org.cloudbus.cloudsim.examples.power.random.RandomRunner


readGraphFile(String) - Method in class org.cloudbus.cloudsim.network.GraphReaderBrite
:   this method just reads the file and creates an TopologicalGraph object

readGraphFile(String) - Method in interface org.cloudbus.cloudsim.network.GraphReaderIF
:   this method just reads the file and creates an TopologicalGraph object

READY - Static variable in class org.cloudbus.cloudsim.Cloudlet
:   The Cloudlet has been assigned to a CloudResource object as planned.

reallocateMigratingInVms() - Method in class org.cloudbus.cloudsim.Host
:   Reallocate migrating in vms.

ReclusteringEngine - Class in org.workflowsim.reclustering
:   A ReclusteringEngine creates a new clustered job after a job fails

ReclusteringEngine() - Constructor for class org.workflowsim.reclustering.ReclusteringEngine


RECORD\_STATISTICS - Static variable in class org.cloudbus.cloudsim.core.CloudSimTags
:   Denotes a request to record events for statistical purposes.

recordList - Static variable in class org.workflowsim.failure.FailureMonitor
:   All the record list.

recvPktlist - Variable in class org.cloudbus.cloudsim.network.datacenter.NetworkVm


redistributeMipsDueToOverSubscription() - Method in class org.cloudbus.cloudsim.VmSchedulerTimeSharedOverSubscription
:   This method recalculates distribution of MIPs among VMs considering eventual shortage of MIPS
    compared to the amount requested by VMs.

REGISTER\_REGIONAL\_GIS - Static variable in class org.cloudbus.cloudsim.core.CloudSimTags
:   Denotes a request to register a CloudResource entity to a regional CloudInformationService
    (GIS) entity

REGISTER\_REPLICA\_CTLG - Static variable in class org.cloudbus.cloudsim.DataCloudTags
:   Registers a Replica Catalogue (RC) entity to a Data GIS

REGISTER\_RESOURCE - Static variable in class org.cloudbus.cloudsim.core.CloudSimTags
:   Denotes a grid resource to be registered.

REGISTER\_RESOURCE\_AR - Static variable in class org.cloudbus.cloudsim.core.CloudSimTags
:   Denotes a grid resource, that can support advance reservation, to be registered.

registerOtherEntity() - Method in class org.cloudbus.cloudsim.Datacenter
:   Overrides this method when making a new and different type of resource.

remove(SimEvent) - Method in class org.cloudbus.cloudsim.core.FutureQueue
:   Removes the event from the queue.

removeAll(Collection<SimEvent>) - Method in class org.cloudbus.cloudsim.core.FutureQueue
:   Removes all the events from the queue.

removeDuplicateMontage() - Method in class org.workflowsim.clustering.VerticalClustering
:   Remove duplicate just for Montage Set in reducer.method

removeLocalFile(File) - Method in class org.workflowsim.CondorVM
:   Removes a file from the local file system

removeMigratingInVm(Vm) - Method in class org.cloudbus.cloudsim.Host
:   Removes the migrating in vm.

renameFile(File, String) - Method in class org.cloudbus.cloudsim.HarddriveStorage
:   Renames a file on the storage.

renameFile(File, String) - Method in interface org.cloudbus.cloudsim.Storage
:   Renames a file on the storage.

ReplicaCatalog - Class in org.workflowsim.utils
:   ReplicaCatalog stores all the data information and where (site) there are

ReplicaCatalog() - Constructor for class org.workflowsim.utils.ReplicaCatalog


ReplicaCatalog.FileSystem - Enum in org.workflowsim.utils
:   File System

REQUEST\_REGIONAL\_GIS - Static variable in class org.cloudbus.cloudsim.core.CloudSimTags
:   Denotes a request to get a list of other regional GIS entities from the system GIS entity

requestclass - Variable in class org.cloudbus.cloudsim.network.datacenter.AppCloudlet


requiresFiles() - Method in class org.cloudbus.cloudsim.Cloudlet
:   Checks whether this cloudlet requires any files or not.

ResCloudlet - Class in org.cloudbus.cloudsim
:   CloudSim ResCloudlet represents a Cloudlet submitted to CloudResource for processing.

ResCloudlet(Cloudlet) - Constructor for class org.cloudbus.cloudsim.ResCloudlet
:   Allocates a new ResCloudlet object upon the arrival of a Cloudlet object.

ResCloudlet(Cloudlet, long, int, int) - Constructor for class org.cloudbus.cloudsim.ResCloudlet
:   Allocates a new ResCloudlet object upon the arrival of a Cloudlet object.

ResCloudletList - Class in org.cloudbus.cloudsim.lists
:   ResCloudletList is a collection of operations on lists of ResCloudlets.

ResCloudletList() - Constructor for class org.cloudbus.cloudsim.lists.ResCloudletList


reserveSpace(int) - Method in class org.cloudbus.cloudsim.HarddriveStorage
:   Makes a reservation of the space on the storage to store a file.

reserveSpace(int) - Method in interface org.cloudbus.cloudsim.Storage
:   Makes a reservation of the space on the storage to store a file.

RESOURCE\_AR\_LIST - Static variable in class org.cloudbus.cloudsim.core.CloudSimTags
:   Denotes a list of hostList that only support advance reservation.

RESOURCE\_CHARACTERISTICS - Static variable in class org.cloudbus.cloudsim.core.CloudSimTags
:   Denotes grid resource characteristics information.

RESOURCE\_CHARACTERISTICS\_REQUEST - Static variable in class org.cloudbus.cloudsim.core.CloudSimTags
:   Denotes request for grid resource characteristics information.

RESOURCE\_DYNAMICS - Static variable in class org.cloudbus.cloudsim.core.CloudSimTags
:   Denotes grid resource allocation policy.

RESOURCE\_LIST - Static variable in class org.cloudbus.cloudsim.core.CloudSimTags
:   Denotes a list of all hostList, including the ones that can support advance reservation.

RESOURCE\_NUM\_FREE\_PE - Static variable in class org.cloudbus.cloudsim.core.CloudSimTags
:   Denotes a request to get the total number of free Processing Elements (PEs) of a resource.

RESOURCE\_NUM\_PE - Static variable in class org.cloudbus.cloudsim.core.CloudSimTags
:   Denotes a request to get the total number of Processing Elements (PEs) of a resource.

RESOURCE\_Register - Static variable in class org.cloudbus.cloudsim.core.CloudSimTags


resourceExist(int) - Method in class org.cloudbus.cloudsim.core.CloudInformationService
:   Checks whether the given CloudResource ID exists or not.

resourceExist(Integer) - Method in class org.cloudbus.cloudsim.core.CloudInformationService
:   Checks whether the given CloudResource ID exists or not.

resourceSupportAR(Integer) - Method in class org.cloudbus.cloudsim.core.CloudInformationService
:   Checks whether a given resource ID supports Advanced Reservations or not.

resourceSupportAR(int) - Method in class org.cloudbus.cloudsim.core.CloudInformationService
:   Checks whether a given resource ID supports Advanced Reservations or not.

restoreAllocation() - Method in class org.cloudbus.cloudsim.power.PowerVmAllocationPolicyMigrationAbstract
:   Restore allocation.

RESUMED - Static variable in class org.cloudbus.cloudsim.Cloudlet
:   The Cloudlet has been resumed from PAUSED state.

resumeSimulation() - Static method in class org.cloudbus.cloudsim.core.CloudSim
:   This method is called if one wants to resume the simulation that has previously been paused.

RETURN\_ACC\_STATISTICS\_BY\_CATEGORY - Static variable in class org.cloudbus.cloudsim.core.CloudSimTags
:   Denotes a request to send an Accumulator object based on category into an event scheduler.

RETURN\_STAT\_LIST - Static variable in class org.cloudbus.cloudsim.core.CloudSimTags
:   Denotes a request to get a statistical list.

ROOT\_LEVEL - Static variable in class org.cloudbus.cloudsim.network.datacenter.NetworkConstants


RootSwitch - Class in org.cloudbus.cloudsim.network.datacenter
:   This class allows to simulate Root switch which connects Datacenter to external network.

RootSwitch(String, int, NetworkDatacenter) - Constructor for class org.cloudbus.cloudsim.network.datacenter.RootSwitch
:   Constructor for Root Switch We have to specify switches that are connected to its downlink
    ports, and corresponding bandwidths

RootSwitchPort - Static variable in class org.cloudbus.cloudsim.network.datacenter.NetworkConstants


run() - Static method in class org.cloudbus.cloudsim.core.CloudSim
:   Start the simulation running.

run() - Method in class org.cloudbus.cloudsim.core.SimEntity


run() - Method in class org.workflowsim.clustering.balancing.BalancedClustering


run() - Method in class org.workflowsim.clustering.balancing.methods.BalancingMethod
:   BalancingMethod is the super class and it should not be used.

run() - Method in class org.workflowsim.clustering.balancing.methods.ChildAwareHorizontalClustering
:   The main function

run() - Method in class org.workflowsim.clustering.balancing.methods.HorizontalDistanceBalancing
:   The main function

run() - Method in class org.workflowsim.clustering.balancing.methods.HorizontalImpactBalancing
:   The main function

run() - Method in class org.workflowsim.clustering.balancing.methods.HorizontalRandomClustering
:   The main function

run() - Method in class org.workflowsim.clustering.balancing.methods.HorizontalRuntimeBalancing
:   The main function

run() - Method in class org.workflowsim.clustering.balancing.methods.VerticalBalancing
:   The main function

run() - Method in class org.workflowsim.clustering.BasicClustering
:   The main function of BasicClustering

run() - Method in class org.workflowsim.clustering.BlockClustering
:   The main function

run() - Method in interface org.workflowsim.clustering.ClusteringInterface
:   the main function.

run() - Method in class org.workflowsim.clustering.HorizontalClustering
:   The main function

run() - Method in class org.workflowsim.clustering.VerticalClustering
:   The main function

run() - Method in class org.workflowsim.planning.BasePlanner
:   The main function

run() - Method in interface org.workflowsim.planning.PlannerInterface
:   the main function.

run() - Method in class org.workflowsim.planning.RandomPlanner
:   The main function

run() - Method in class org.workflowsim.scheduler.BaseScheduler
:   The main function

run() - Method in class org.workflowsim.scheduler.DataAwareScheduler


run() - Method in class org.workflowsim.scheduler.FCFSScheduler
:   The main function

run() - Method in class org.workflowsim.scheduler.MaxMinScheduler


run() - Method in class org.workflowsim.scheduler.MCTScheduler


run() - Method in class org.workflowsim.scheduler.MinMinScheduler


run() - Method in interface org.workflowsim.scheduler.SchedulerInterface
:   the main function.

run() - Method in class org.workflowsim.scheduler.StaticScheduler


runClockTick() - Static method in class org.cloudbus.cloudsim.core.CloudSim
:   Internal method used to run one tick of the simulation.

RUNNABLE - Static variable in class org.cloudbus.cloudsim.core.SimEntity
:   The Constant RUNNABLE.

RunnerAbstract - Class in org.cloudbus.cloudsim.examples.power
:   The Class RunnerAbstract.

RunnerAbstract(boolean, boolean, String, String, String, String, String, String) - Constructor for class org.cloudbus.cloudsim.examples.power.RunnerAbstract
:   Run.

running() - Static method in class org.cloudbus.cloudsim.core.CloudSim
:   Check if the simulation is still running.

runningCloudlets() - Method in class org.cloudbus.cloudsim.CloudletScheduler
:   Returns the number of cloudlets runnning in the virtual machine.

runningCloudlets() - Method in class org.cloudbus.cloudsim.CloudletSchedulerSpaceShared
:   Returns the number of cloudlets runnning in the virtual machine.

runningCloudlets() - Method in class org.cloudbus.cloudsim.CloudletSchedulerTimeShared
:   Returns the number of cloudlets runnning in the virtual machine.

runningCloudlets() - Method in class org.cloudbus.cloudsim.network.datacenter.NetworkCloudletSpaceSharedScheduler
:   Returns the number of cloudlets runnning in the virtual machine.

runStart() - Static method in class org.cloudbus.cloudsim.core.CloudSim
:   Internal method used to start the simulation.

runStop() - Static method in class org.cloudbus.cloudsim.core.CloudSim
:   Internal method used to stop the simulation.

A B C D E F G H I J L M N O P Q R S T U V W Z

- Overview
- Package
- Class
- Use
- Tree
- Deprecated
- Index
- Help

- Prev Letter
- Next Letter

- Frames
- No Frames

- All Classes
